# A metadata-aware application for remote scoring and exchange of tissue microarray images

**DOI:** 10.1186/1471-2105-14-147

**Published:** 2013-05-01

**Authors:** Lorna Morris, Andrew Tsui, Charles Crichton, Steve Harris, Peter H Maccallum, William J Howat, Jim Davies, James D Brenton, Carlos Caldas

**Affiliations:** 1 Department of Oncology, University of Cambridge and Cancer Research UK Cambridge Research Institute, Li Ka Shing Centre, Cambridge, CB2 0RE, UK; 2 Cambridge Experimental Cancer Medicine Centre, Li Ka Shing Centre, Cambridge, CB2 0RE, UK; 3 Addenbrooke’s Hospital, Cambridge University Hospital NHS Foundation Trust and NIHR Cambridge Biomedical Research Centre, Cambridge, CB2 2QQ, UK; 4 Department of Computer Science, University of Oxford, Wolfson Building, Parks Road, Oxford, OX1 3QD, UK

**Keywords:** Tissue microarray, TMA, Metadata, XML, Software

## Abstract

**Background:**

The use of tissue microarrays (TMA) and advances in digital scanning microscopy has enabled the collection of thousands of tissue images. There is a need for software tools to annotate, query and share this data amongst researchers in different physical locations.

**Results:**

We have developed an open source web-based application for remote scoring of TMA images, which exploits the value of Microsoft Silverlight Deep Zoom to provide a intuitive interface for zooming and panning around digital images. We use and extend existing XML-based standards to ensure that the data collected can be archived and that our system is interoperable with other standards-compliant systems.

**Conclusion:**

The application has been used for multi-centre scoring of TMA slides composed of tissues from several Phase III breast cancer trials and ten different studies participating in the International Breast Cancer Association Consortium (BCAC). The system has enabled researchers to simultaneously score large collections of TMA and export the standardised data to integrate with pathological and clinical outcome data, thereby facilitating biomarker discovery.

## Background

Tissue microarrays (TMAs) enable the analysis and visualisation of hundreds of tissues on a single slide, resulting in the conservation of tissue and the reduction of inter-experimental variability. Multiple cylindrical cores (usually less than 1 mm in size) are cut from donor tissue blocks and placed in a recipient block to create an array [1]. Sections from the recipient block therefore contain discs of tissue, each representing the morphology of the original block, which can be analysed by immunohistochemistry (IHC) using antibodies to detect a panel of candidate biomarkers. The subsequent manual scoring of the TMA slides via a microscope can be an error-prone and time-consuming activity.

Recent advances in automated scanning microscopy have enabled the rapid acquisition and digitisation of slides. The Leica Ariol automated scanning system captures and analyses TMA slides and stores the individual core images and associated metadata in a proprietary database [2]. This data must then be extracted and presented in a form more amenable to analysis, annotation, and access by different researchers.

The volume of image data generated from scanning large collections of TMA slides represents a challenge in terms of storage, transfer, and analysis. Although automated techniques for the quantification of staining in images are being developed [3] manual scoring is considered the ‘Gold Standard’. This is facilitated by the simultaneous presentation of large numbers of images for inspection and annotation; this is particularly helpful in dealing with variations in the quality of staining. It is facilitated also through remote access across institutions, allowing experts to collaborate: promoting standardisation and allowing for validation. Pooling data from multiple institutions or studies requires the data to be standardised so that scores can be compared and integrated. Pooled data sets provide a larger sample size for statistical analysis with a greater statistical power than analysing the smaller number of scores collected from a single study.

Commercial applications such as Slidepath and PathXL [4,5] address several aspects of TMA data management, including on-line scoring of TMA images. These applications are, however, intended as complete, closed solutions: users are not expected or permitted to rewrite aspects of the software in order to support existing laboratory workflows. Even where the software is a good match for local processes, the licensing and support costs can be prohibitive.

Stanford University have developed TMAD, a public resource for disseminating annotated tissue images and associated expression data [6], where they have addressed a wide functionality including: public access; TMA design; support for on-line and microscope scoring; browsing and incorporation of a flexible data analysis (e.g. hierarchical clustering) pipeline.

Other efforts for management of TMA data include the Virtual Tissue Matrix and TMAJ [7,8]. The former focuses on facilitating on-line review of TMAs, with the facility to zoom and pan around TMA core images and associate a score, which is stored in a relational database. In TMAJ the user can enter pathologic data such as the histological type, the values of which are constrained according to the tissue in the displayed image. Further customisation of the user interface may be required to allow users to enter data for different types of scoring systems e.g. numerical ranges to describe staining intensity. Some of the existing tools, for example TMAD and TMAJ present images for scoring in a fixed order and magnification and thus the scorer cannot navigate around the array as they would with a microscope. This is an important feature of Slidepath software and a requirement for the pathologists using our system.

Viti et al. [9] address the integration of clinical data with TMA data by use of ontologies for example the Gene Ontology (GO). Storing data according to agreed standards is important for the sharing, re-analysis and archiving of the rapidly increasing volumes of TMA image data. Microarray Gene Expression – Markup Language (MAGE-ML) system is an established standard for the description of microarray gene expression experiments [10]. Many of the public databases for the storage of microarray data require data submitted to be in MAGE-ML format. Whilst MAGE-ML represents the format of data for exchange, MIAME (Minimum Information About a Microarray Experiment) describes a checklist of data required in order for an independent researcher to be able to interpret the results of the experiment unambiguously and potentially to reproduce the experiment.

**Figure 1:**
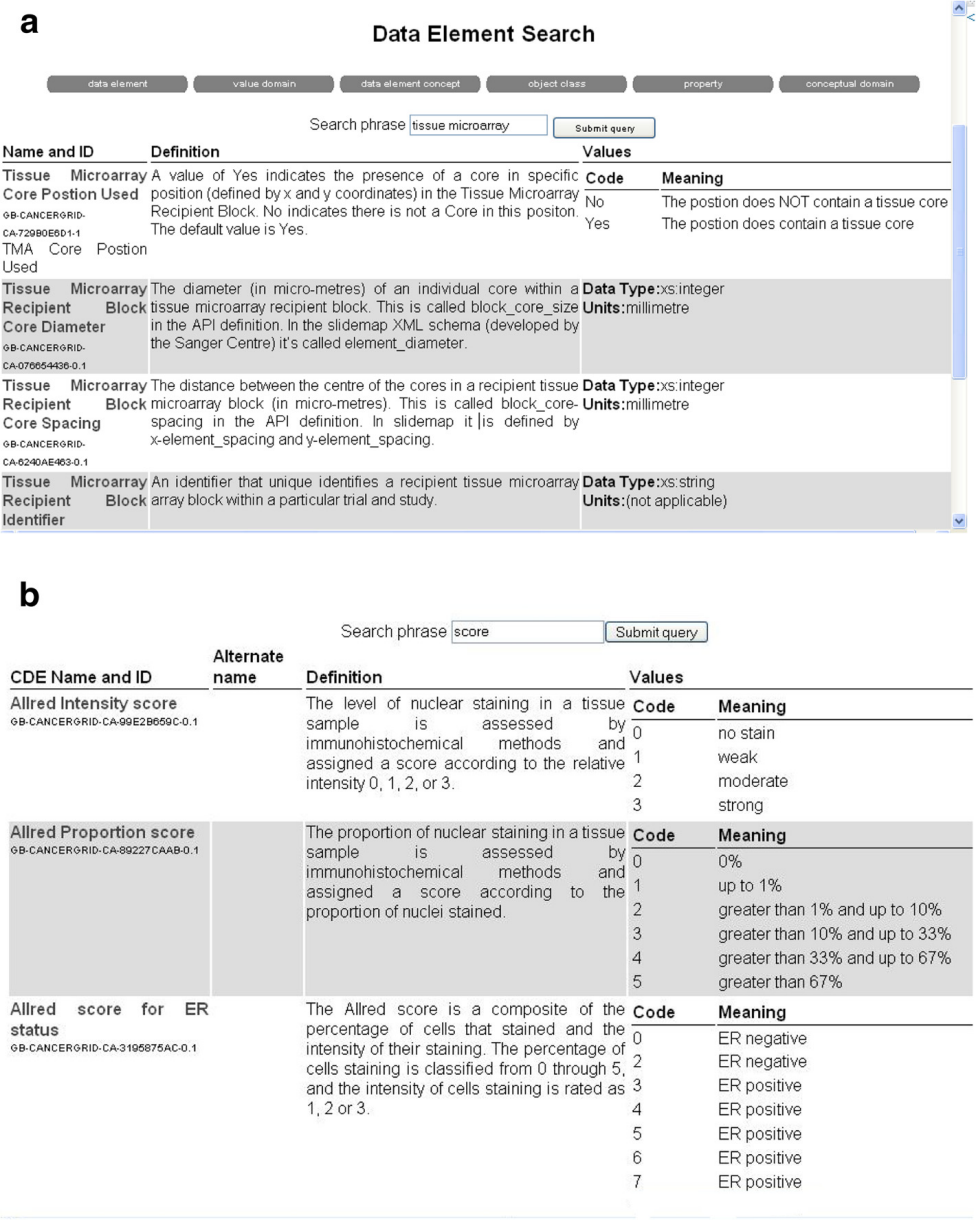
**Screenshots of the CancerGrid metadata registry.** CDE definitions for (**a**) attributes (XML elements) used in the slidemap schema (**b**) attributes describing specific scoring systems used in the TMA results schema.

There has been some effort to establish a standard for TMA data. The Association for Pathology Informatics (API) Extensible Mark-up Language (XML) TMA Data Exchange Specification (TMA DES) proposed in April 2003 provides a community-based, open source tool for sharing TMA data in a common format [11]. This defines a set of Common Data Elements (CDEs) to describe TMA data. A Common Data Element (CDE) is a metadata definition with an informal explanation of its meaning and usage, a list of alternative names and definitions, units of measurement, and the type of values to be recorded ([12], for examples see Table 1 and Figure 1). The TMA DES schema is flexible so that only a subset of the CDEs defined in the standard are required to constitute a valid TMA DES file.

**Table 1:** Comparison of cancergrid common data elements (CDEs) with CDEs from the association of pathology informatics data exchange specification (DES)

**Cancergrid CDE name**	**API DES XML tag name**	**Comments**
Donor Tissue Block Identifier	core_histo-repository_donor-block	
Tissue Microarray Recipient Block Identifier	block_identifier	
Tissue Microarray Recipient Block Core Diameter	block_core_size	
Tissue Microarray Recipient Block Core Spacing	block_core_spacing	
Tissue Microarray Recipient Block Core Position Used		Used to indicate a gap in the TMA Recipient Block where a core has not been inserted.
Tissue Microarray Recipient Block Slice Number		Useful for querying whether a core image is from a slice adjacent to another core image.
Tissue Microarray Recipient Block x coordinate		block_array-hash describes the x,y location for each core in the block array, associating an identifier with each core location.
Tissue Microarray Recipient Block y coordinate		
Tissue Microarray Slide Identifier	slide_identifier	
Tissue Microarray Core Element Identifier		core_array_id serves the same function, to uniquely identify a core within a TMA Recipient Block
Allred Intensity Score		core_results_tissue-intensity is a more general numerical value to define the relative intensity of staining
Allred Proportion Score		Represents a specific staining system.
Allred Score for ER status		
Percentage tissue staining	core_results_percent-tissue-staining	
HER2 status by immunohistochemistry		Represents a specific staining system.
Binary biomarker staining result		A simple scoring system with a binary value to indicate whether the tissue is positively or negatively stained for a particular marker.

Several of the existing TMA databases are compatible with this standard. For example, the Stanford TMAD enables the export of data in TMA DES format [6]. TmaDB, based at the University of Leeds is a central repository for archiving all aspects of TMA and associated pathology data on a range of different cancer types and is designed to incorporate many of the TMA DES CDEs [13].

Here we describe an application for the management and storage of TMA images and the associated metadata. The application enables the user to navigate the core images within a slide, zoom and pan around an image, and enter a score constrained to a specific scoring system. It facilitates navigation of thumbnail images in any collection, such as a series of core images that have been selected from a variety of studies for validation of an automated scoring algorithm. We utilise standard definitions from the TMA DES standard and we extend these definitions to describe positional data and specific scoring systems. The workflow incorporates TMA images captured using the Leica Ariol image scanning microscope [2], but is compatible with JPEG images captured using different scanners. The emphasis throughout is upon ease of use and interoperability; the intention is that the application should be used for the specific purpose of image presentation and annotation, exchanging data with other platforms and tools using established XML-based data standards.

## Implementation

We set out to design a database to track donor tissue blocks within TMA slides and create a simple interface for on-line scoring and sharing of TMA images and associated data, to enable the scoring data to be linked to the related patient data.

### Creation of common data elements

We looked at the data exchanged between 2 laboratories (Strangeways Research Laboratory (SRL) and the CRI CRUK Histopathology Core Facility (CRI)). SRL create and section TMAs in preparation for IHC, scanning and analysis at the CRI. Slides are scanned using the Leica Ariol system, before being associated with a score by a pathologist. There is an overlap between the TMA DES standard and the data required for the slidemap schema used by the Ariol system. We used this common set to define a minimum set of CDEs to describe the layout of a tissue microarray, in order that it could be interpreted and processed by both laboratories.

We also defined a further set of CDEs that describe the results of IHC on a TMA slide using several widely used scoring systems (for example the Allred score for ER status [14]). Each CDE gives a precise definition with a set of allowed values, making the data explicit so that it can be understood and utilised in other software systems and by other researchers. The CDEs created are compared with the TMA DES definitions in Table 1. Where there is no direct equivalent the comments indicate why we created alternative or extended definitions.

The CDEs were created using the CancerGrid Metadata Repository (MDR), via the web-based interface used to specify the definition, value domain and units of measure [12,15,16]. The resulting CDEs are stored in XML format, the structure conforming to the ISO/IEC11179 international standard for metadata repositories [17]. The MDR provides tools for registering, updating and browsing CDEs, concepts, properties and their definitions, as well as searching and basic classification tools. Figure 1 shows examples of CDEs created to describe TMA layout and to define Allred score.

### Data model and database

**Figure 2:**
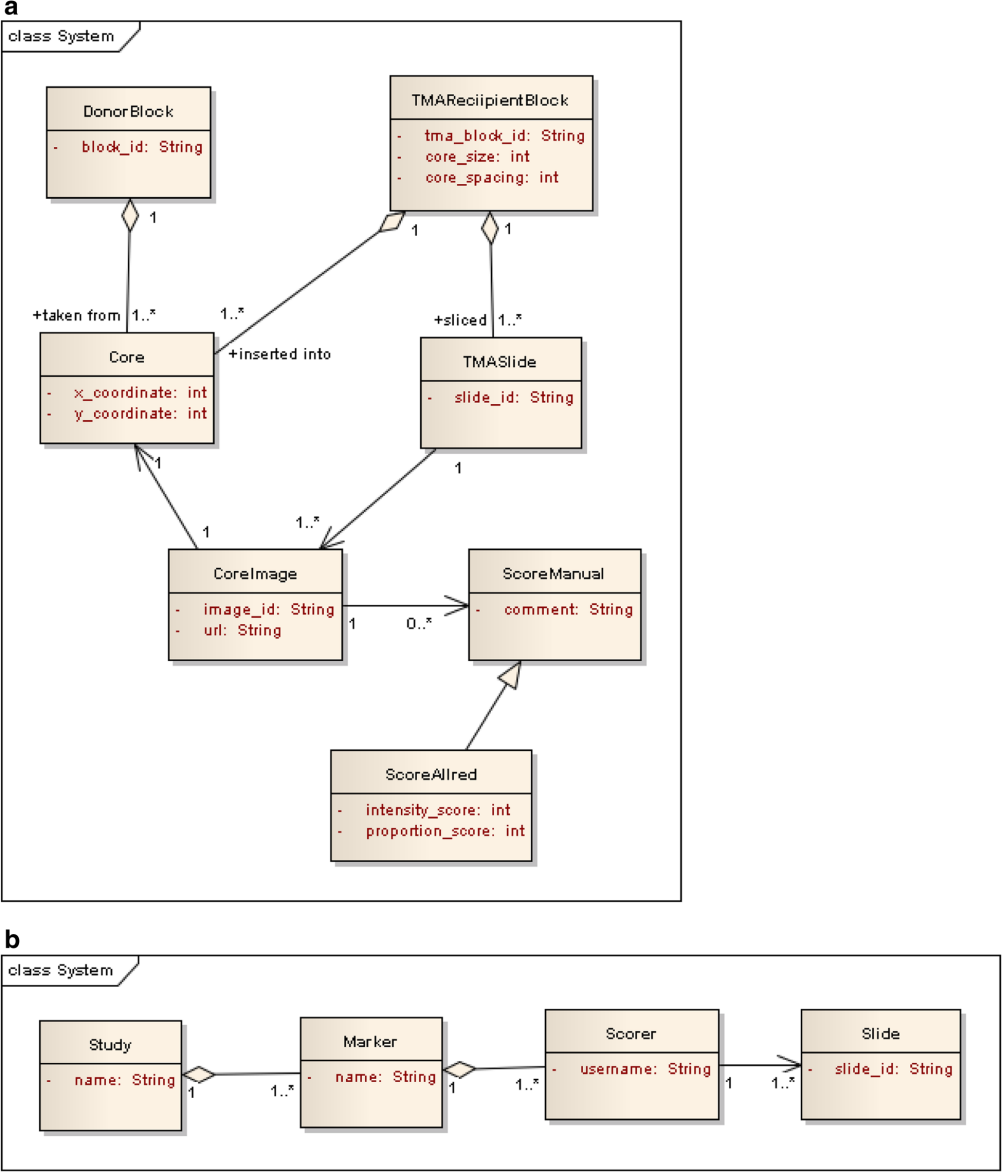
**Schematic models for Cancergrid TMA database.** (**a**) Class model to show the relationships between the entities required for tracking and scoring TMA core images within a ‘TMASlide’. The model shows the data used to track the scores for a specific image back to the ‘DonorBlock’, associated with an individual patient. Patient details are not stored in the database. This is a conceptual model from which the XML schema is derived. The database stores XML files corresponding to this schema, which specify the relationship between ‘TMASlide’, associated core images, coordinates of associated ‘Cores’ and a set of manual scores. ScoreAllred represents an Allred score, a type of ScoreManual. Aggregation relationships between ‘DonorBlock’, ‘Core’ and ‘TMARecipientBlock’, show that one or more ‘Cores’ are taken from a ‘DonorBlock’ and a TMARecipientBlock is composed of many ‘Cores’ (these relationships are specified in the Genetix slidemap schema). (**b**) The database collection hierarchy. XML files (‘TMASlide’) are associated with a particular ‘Scorer’ (a pathologist with a username a password to login to the system). The ‘Scorer’ collection is part of the ‘Marker’ collection (slide stain) and there can be multiple ‘Marker’ collections associated with a particular study.

We created a class model to capture the data and relationships between the main entities required in our TMA pipeline (Figure 2a). Each entity contains one or more attributes defined by a CDE. The model ensures that individual scores in TMA slides can be related back to a patient from whom the tissue was derived. Our database does not contain information about sample collection. Institutions have their own systems for tracking of tissue blocks and therefore our focus was the tracking and scoring of images within the TMA.

Our pipeline has been used to capture TMA data from ten international institutions collaborating in Breast Cancer Association Consortium (BCAC). BCAC was established in 2005 to conduct such collaborative studies in breast cancer [18,19]. The majority of these institutions use Microsoft Excel to specify the positions of cores within an array. The Ariol software requires positional information to be defined in an XML file (slidemap) conforming to a specific schema. In order to convert Excel data files to this format we employ XML transformation (XSLT) to transform these files into slidemap files that can be utilised by the Ariol system.

We use the open source eXist XML database [20]. Our database is structured according to hierarchical collections (Figure 2b). The root collection is study, which corresponds to a set of tissues collected for a specific study or clinical trial. Each study collection is split into several sub-collections, a collection for each biomarker that has been assessed and further sub-collections for each user. At the base of the hierarchy are the sets of individual XML files, each file representing a slide scored by a single user or generated from an automated analysis.

### Image export and batch conversion of JPEG images to “deep zoom” images

Leica provide a web service programming interface for Ariol, which enables the export of images and metadata from their database to be automated. A Java web service client was written to export the core images (JPEG format) and associated metadata (XML) for each individual TMA slide. The JPEG images are exported at 70% resolution, which according to feedback from multiple pathologists was a suitable resolution for manual scoring. Images are renamed with a unique identifier combining the slide identifier and × and y coordinates (slideID_×_y_version.JPEG).

Microsoft Silverlight “Deep Zoom” [21] was used to create “Deep Zoom” images of TMA cores. Deep Zoom enables interactive viewing of high-resolution images, allowing the user to zoom in and out of images rapidly without affecting the performance of the application. This was of critical importance, as large collections of images need to be downloaded and viewed in high resolution. A batch converter was written in C#, to automate conversion of large collections of JPEG images into Deep Zoom format. The images were also cached on the client to enable rapid access to slides upon subsequent viewing. A demonstration version of the Image Scorer application supports touch interaction to allow users with touch screen monitors to zoom into and out of images with the pinch gesture.

The batch image converter and the web service Client are written as separate applications. It is useful to have the batch converter as a separate service to enable the conversion of images from different scanners to be incorporated into the pipeline.

**Figure 3:**
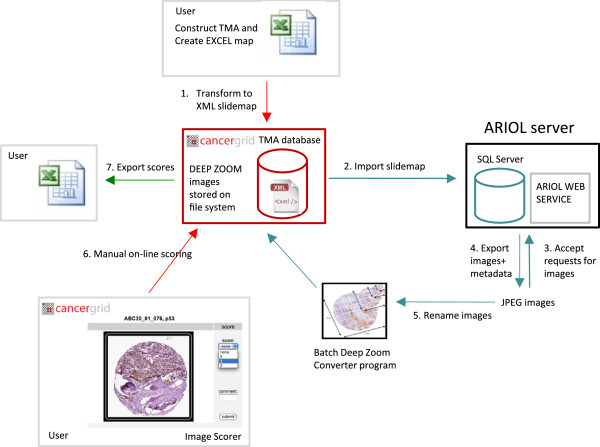
**Workflow for Cancergrid TMA pipeline.** The workflow shows the relationship between software components and data flow between the components. **Red arrows** indicate user input; **blue arrows** indicate internal data flow between the Cancergrid TMA database, the export of images and metadata from ARIOL and image conversion to prepare images for the Image Scorer. The image service application requests images from ARIOL (Ariol Web Service client), converts images into Deep Zoom format (Batch Deep Zoom Converter), generates a score form template for the slide corresponding to the core images exported and uploads the resources to the eXist server. The Cancergrid TMA database consists of eXist installed within Tomcat and xquery and XSLT for generate the web forms and Microsoft Silverlight for displaying the images. **Green arrow** indicates export of TMA scores from the database.

The workflow in Figure 3 shows the relationship between software components and the flow of data in the TMA pipeline, from user input to export of scoring data.

### Web application design

The user interface was written using a combination of xquery, XSLT and Silverlight. Ariol export generates an XML file for each scanned slide, which includes x and y coordinates and donor block identifiers from the slidemap and output from any automated analysis that has been carried out. We transform the XML file (using XSLT) to extract the data to create a web form template for manual scoring. The XML template is stored in the eXist database and by making use of the xquery update extensions of eXist we store scores from user input.

## Results and discussion

The main challenges in designing our application were to create a database structure to enable the tracking of individual tissues cores within an array and to ensure that multiple users could navigate and score images via the Internet. The tracking requirement was necessary to ensure scores could be associated with clinical data from the corresponding patient. Scoring of images by researchers in multiple institutions ensures that analysis can be standardised and validated. The volume of images being processed through our pipeline makes a high-throughput approach essential.

The starting point for scoring a TMA in the Cancergrid Image Scorer is to login via the web interface. We make use of https and eXist database user management to control access to slides in different collections for different users.

**Figure 4:**
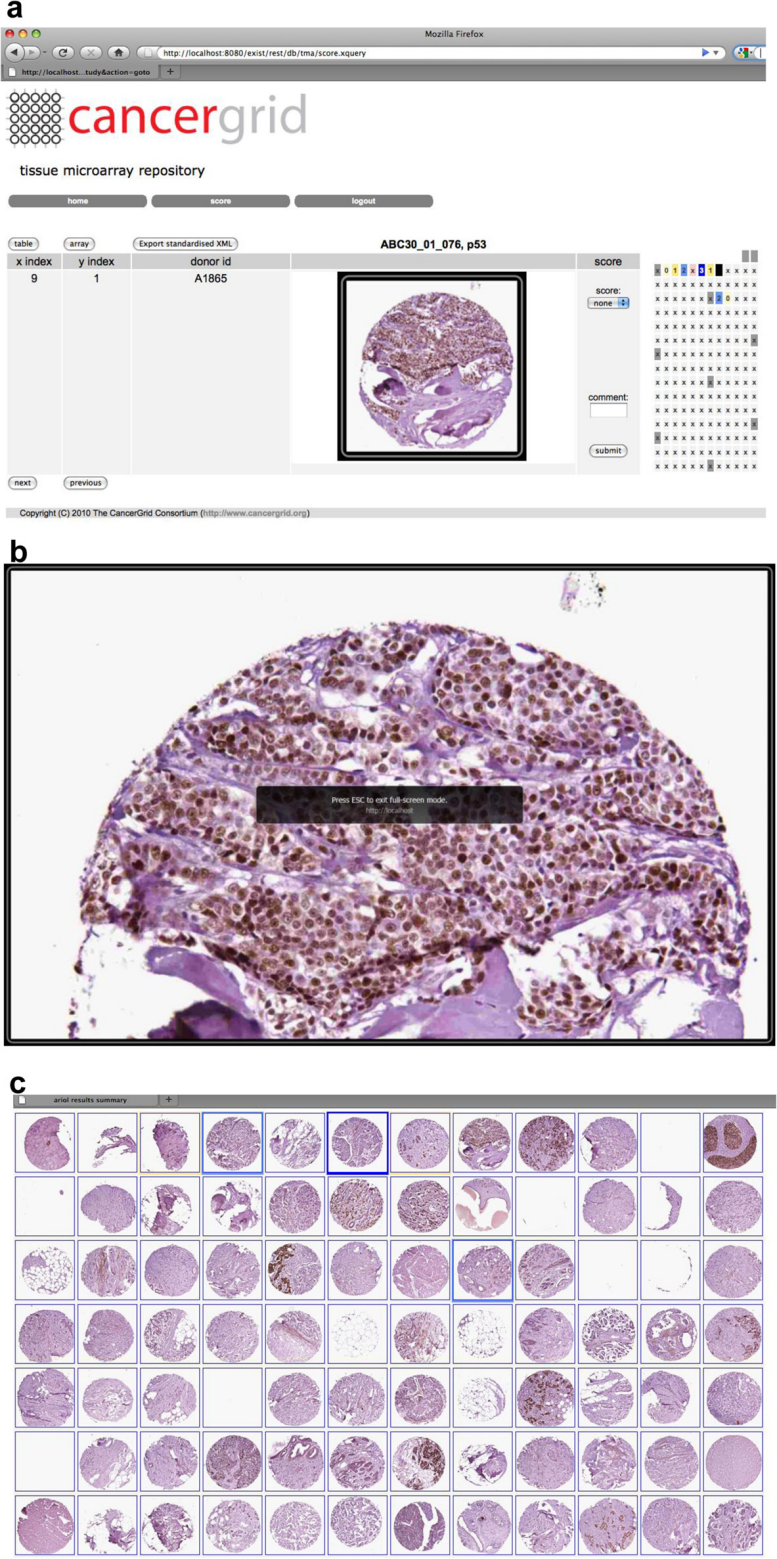
**Screen shots of Image Scorer web application.** (**a**) Heatmap view; shows a thumbnail of a TMA core stained for ER and a heatmap representation of the TMA slide, coloured according to intensity values. In the example shown the scoring system is a range of 0-3 inclusive (0=pale yellow; 1=intense yellow; 2=pale blue; 3=intense blue). Grey squares represent marker cores used for array orientation, pink represent no tumour. The black square indicates the position in the array of the current image. (**b**) Zoomable view showing individual core image in full screen mode. The user can pan around the image and zoom into and out of it. (**c**) Array view, showing layout of thumbnail images on the virtual slide. Clicking on a thumbnail returns the user to the web form for entering the score.

The user can then select a particular study of interest and then filter this by available biomarkers and finally select a particular slide for scoring. This brings up a thumbnail image of the first (top left) core in the array and an associated scoring form, with a heatmap representation of the slide on the right-hand panel (Figure 4a). Clicking on the thumbnail brings up a full screen image, which the user can zoom into and pan around to explore the core in greater detail (Figure 4b). The heatmap is colour-coded according to the intensity value the user has submitted for each core image and the current position of the selected core is represented by a black square. The heatmap enables the user to quickly navigate the TMA, core by core, keep track of their progress and enter their scores into the database.

An alternative array view of the slide, with the thumbnail images arranged according to their co-ordinates, assembled into a virtual TMA slide is also available (Figure 4c). The system has also been used to score “virtual” TMA views, that is a grid-view containing different TMA core images from multiple TMAs. This has proved effective for manual on-line review, for the validation of new automated scoring algorithms.

The heatmap view has been useful for training, allowing easy review of scores after consultation with an experienced pathologist. For example checking Allred scores with values 2 and 3; these borderline scores distinguish between ER positive and ER negative breast cancer tumours in diagnosis [14]. The heatmap view is also a useful visualisation to enable comparison between different users scores side-by-side to investigate discrepant scores and to reveal slide defects such as uneven staining across the slide.

Scores for a particular slide can be viewed in tabular format and can be imported into software tools such as Excel or SPSS for survival analysis. Each row in the exported table contains a unique identifier, which links the TMA core image back to the patient from which the tissue was derived. The export TMA DES button enables download in this standard XML format [11] to allow integration into other software implementing this standard.

A further advantage of using an XML-based approach is that XSLT can be used to create different views of the data. For example we present the data as an array, heatmap and in tabular form. This gives a clear separation between the data and the presentation layer. XML is self-describing and as such is flexible enough to be imported into future pipelines using new types of software.

Our application could be used to score TMA slides from any scanning microscope system, if the pre-requisite XML template file and set of individual core images (JPEG) are provided. Pattern recognition software for splitting full-slide TMA scans into individual core images is now available from a variety of vendors including Ariol, Aperio and Slidepath [2,4,22]. Our database does not include clinical data as, due to the multi-institutional nature of this research, very often this may be housed elsewhere. In addition a research laboratory may not have the permission or ethical approval to database clinical trial data, especially when it contains patient details. We did not aim to create a monolithic data warehouse to store all of this data in one place, rather our approach was to create a system whereby data could be easily shared amongst collaborating institutions.

We considered using and adapting other open source tools such as the TMAJ and Virtual Tissue Matrix [7,8]. Although these appeared to be excellent tools for the purpose of scoring TMA images we felt we gained additional benefits by using Microsoft Silverlight to enable us to build a rich web-based interface. Furthermore by using an XML-based approach and defining CDEs to add semantics to our data we ensure that the exported data can be described by a set of externally referenced definitions, which add meaning to the data outside the context of the database. Employing CDEs from existing standards provides a means to document the data collected and ensure that the pathologists in different institutions are all using the same values to describe the same term. They also enable the data to be re-used or analysed using new or yet to be developed methodologies.

Standardised scoring systems exist e.g. Allred [14] but there are also a variety of ad hoc scoring systems used to assess the level of particular markers in different contexts, and the use of scoring systems can vary in different laboratories and can depend on the research question being asked. The recent proposed extension to TMA DES aims to allow the sharing of existing scoring systems, thus improving agreement between studies [23].

Future work will focus on adding query functionality to the web interface. For example, the ability to view all TMA core images from the same donor block (i.e. patient) stained with antibodies for different biomarkers. The Cancergrid MDR was used to create metadata definitions of our data, each CDE giving a precise definition with a set of allowed values. These allowed values are utilised in drop-down menus to constrain user input and ensure the data is valid. Making these definitions available as prompts in the web form could enhance the user interface. Currently the creation of forms for different scoring systems is performed in an ad-hoc way by implementing the appropriate XML schema and creating the corresponding web-forms in HTML and xquery. The Cancergrid project has created a toolset for model driven generation of case report forms for clinical trials [15]. Form editing could be unified with these tools to enable users to create and edit their own scoring forms.

We have implemented support for touch interaction, as there is a growing interest in this technology. The use of touch control screens is particularly suitable for annotating regions within an image, for example segmenting images into tumour and non-tumour. Deployment of our software on portable tablet computers to provide flexibility for when and where the pathologist reviews images would be a useful future development.

## Conclusions

The software application we have described here has been used to automate a workflow for the high-throughput manual scoring of thousands of TMA images and make the results available for integration with clinical data. We have used the application to collect manual scores for TMAs for several Phase III breast cancer clinical trials at CRUK Cambridge Research Institute. Furthermore the tool has been utilised to score a subset of images from ten different International (European and USA) studies for quality assurance purposes and comparison with results from different automated scoring algorithms. Our approach has improved the scalability and throughput of translational studies, reducing the dependence on manual processes, which are often error-prone and time-consuming.

The Cancergrid Image Scorer is an open-source product, which exploits the value of a commodity, commercial platform, Microsoft Silverlight. The client requires only the Silverlight browser plug-in, which is freely available for all supported platforms and browsers. The application combines several features, such as the ability to zoom and pan around images, rapidly navigate and score individual core images and allow export of data in a standardised format. It has been shown to be a valuable tool for the sharing of images and scores across multiple sites.

By linking our data to standard definitions, captured as metadata, we make it easier for others to understand, re-use, and validate our work. These links can be exploited automatically in subsequent analysis and visualisation, customising the behaviour of data management and processing tools to suit the precise nature of the data collected. Scalable, reproducible, computer-supported science and medicine will be increasingly dependent upon tools that are metadata-aware: that can create and follow links to descriptions of provenance, context, and interpretation. It is our hope that this tool is a useful contribution to this evolutionary development.

## Availability and requirements

**Project name**: cancergrid-tma

**Project home page**: http://sourceforge.net/projects/cancergrid-tma/

**Operating system(s)**: Windows 7 and XP, Linux, Mac OS X 10.4 or later.

**Programming language**: XQuery/XSLT

**Other requirements**: Java6 “Java SE 6”; eXist (http://exist.sourceforge.net); Microsoft Silverlight 4

**License**: BSD

The manual and README file available from the project homepage describes how to install and setup the software. The Deep Zoom Image Converter is available from the http://www.cancergrid.org web-site (access through the downloads section from the ‘File Repository’ link from the main menu).

## Abbreviations

CDE: Common data element; JPEG: Joint photographic experts group; MAGE-ML: Microarray gene expression – markup language; MDR: Metadata repository; TMA: Tissue microarray; TMA DES: Tissue microarray data exchange specification; XML: eXtensible markup language

## Competing interests

The authors declare that they have no competing interests.

## Authors’ contributions

LM wrote the first draft of the article, created the CDEs, implemented the Ariol web service client, and designed the database model, eXist database structure and implemented the xquery web application. AT implemented the batch Image Converter for creation of the Deep Zoom Images. AT wrote the Javascript code to embed the Silverlight viewer into the eXist web application. SH implemented the Cancergrid MDR and implemented xquery library files. C Crichton and PM discussed the CDE approach for database design and provided input on the chosen approach throughout the design and implementation phases. CCaldas, WH and JB have reviewed the CDEs. JD, JB and CCaldas were responsible for overall project design and supervised the research. All authors have reviewed and edited all drafts of the manuscript. All authors read and approved the final manuscript.
